# Architecture of the superintegron in *Vibrio cholerae*: identification of core and unique genes

**DOI:** 10.12688/f1000research.2-63.v1

**Published:** 2013-02-27

**Authors:** Michel A Marin, Ana Carolina P Vicente

**Affiliations:** 1 Laboratory of Molecular Genetics of Microorganisms, Oswaldo Cruz Institute (IOC), Rio de Janeiro, 4365, PO Box 926 CEP 21045-900, Brazil

## Abstract

**Background: **** *Vibrio* ** *cholerae*, the etiologic agent of cholera, is indigenous to aquatic environments. The *V. cholerae* genome consists of two chromosomes; the smallest of these harbors a large gene capture and excision system called the superintegron (SI), of ~120 kbp. The flexible nature of the SI that results from gene cassette capture, deletion and rearrangement is thought to make it a hotspot of *V.* ** *cholerae* diversity, but beyond the basic structure it is not clear if there is a core genome in the SI and if so how it is structured. The aim of this study was to explore the core genome structure and the differences in gene content among strains of *V. cholerae*.

**Methods: **From the complete genomes of seven *V.* ** *cholerae *and one *Vibrio mimicus* representative strains, **we recovered the SI sequences based on the locations of the structural gene *IntI4* and the *V.* ** *cholerae* **repeats. Analysis of the pangenome, including cluster analysis of functional genes, pangenome profile analysis, genetic variation analysis of functional genes, strain evolution analysis and function enrichment analysis of gene clusters, was performed using a pangenome analysis pipeline in addition to the R scripts, splitsTree4 and genoPlotR.

**Results and conclusions: **Here, we reveal the genetic architecture of the *V. cholerae* SI. It contains eight core genes when *V. mimicus* is included and 21 core genes when only *V. cholerae *strains are considered; many of them are present in several copies. The *V. cholerae* SI has an open pangenome, which means that *V. cholerae* may be able to import new gene cassettes to SI. The set of dispensable SI genes is influenced by the niche and type species. The core genes are distributed along the SI, apparently without a position effect.

## Introduction

**Figure 1.**
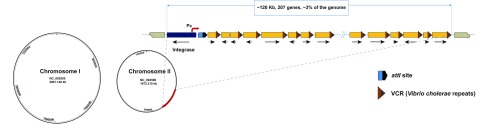
Schematic organization of the *Vibrio cholerae* genome and the superintegron (SI). The functional platform of the SI consists of an integrase gene, a cassette promoter (Pc), and a primary recombination site ( *attI*). The system maintains an array of several cassettes, which generally consist of a promoterless ORF flanked by two recombination sites termed VCR ( *V. cholerae* repeats).

*Vibrio cholerae* is a diverse, environmental, gram-negative bacterial species that can be pathogenic and can cause cholera, a severe diarrheal disease that occurs most frequently in epidemic form ^1, 2^. The *V. cholerae* genome consists of two chromosomes. The largest chromosome of 2.96 Mbp encodes most essential genes. The 1.07 Mbp small chromosome contains few essential genes and the superintegron (SI), a large gene capture and excision system of ~120 kbp ^2^ ( Figure 1). The SI is characterized by a site-specific integrase gene ( *IntI4*) closely associated with a cognate recombination site *attI* and a promoter Pc followed by a large array of gene cassettes. Within the SI, the gene cassettes generally consist of a promoterless open reading frame (ORF) flanked by two recombination sites termed *V. cholerae* repeats (VCRs) ^3^. Cassettes can be excised from any position in the array through VCR × VCR recombination mediated by the integrase. The resulting circular intermediate can then be integrated, preferentially through *attI* × VCR recombination by the integrase, bringing the cassette under control of Pc ^4, 5^. Since gene cassettes are usually promoterless, only the first few cassettes are expressed by Pc and the rest of the array can be seen as a reservoir of standing genetic variation ^5^.

The functions of the majority of the SI genes are unknown; however, a few genes have been characterized and it has been suggested that they are involved in adaptive functions such as toxin-antitoxin (TA) loci. TA loci consist of two genes in an operon encoding a ‘toxin’ and an ‘antitoxin’. The expression of the toxins reduces cell growth and prevents colony formation, thus exerting a bacteriostatic rather than bacteriocidal condition. However, cell viability can be rescued by later overproduction of the cognate antitoxins ^6^.

The pangenome describes the complete repertoire of genes in a bacterial species, which includes the "core genome" containing genes present in all strains, a "dispensable genome" containing genes present in two or more strains, and "unique genes" specific to single strains ^7^. Previous phylogeographic analysis, considering *V. cholerae* strains and its sister species *Vibrio metecus* ^8^, showed that, in contrast to the core genome, the SI displays strong geographical differentiation, and cassettes from the *V. cholerae* group cluster with those of *V. metecus* from the same place rather than with cassettes from geographically distinct *V. cholerae*. It suggested that SI structure is influenced by geographic boundaries and in response to environmental conditions. The flexible nature of the SI that results from gene cassette capture, deletion and rearrangement is thought to make it a hotspot of *V. cholerae* diversity, but beyond the basic structure it is not clear if there is a core genome in the SI and if so how it is structured. The aim of this work was to explore the core genome structure and the differential gene content among strains of *V. cholerae*.

## Methodology

Based on the complete genomes of seven *V. cholerae* and one *V. mimicus* representative strains ( Table 1), we searched repeats above 10 nucleotides and used one VCR sequence (AAC AAA CGC CTC AAG AGG GAC TGT CAA CGC GTG GCG TTT CCA GTC CCA TTG AGC CGT GGT GGT TTC GGT TGT TGT GTT TGA GTT TAG TGT TAT GCG TTG TCA GCC CCT TAG GCG GGC G) to search for sequences with more of 45% nucleotide identity. The SI sequences were recovered using the locations of the structural gene *IntI4* and VCRs identified with the UGENE software ^9^. Cluster analysis of functional genes was performed using the pangenome analysis pipeline ^10^, which searches for homologs or orthologs among multiple genomes using the MultiParanoid (MP) method (based on a 90% nucleotide identity threshold). For each pair of genes in the same cluster, the local matched region is no less than 25% of the longer protein coding sequence and the global matched region is no less than 50% of the longer protein coding sequence. The minimum score value and E-value in BLAST are 50 and 1e-8 ^10^. The gene content was converted to a presence/absence (0/1) matrix and then the core, dispensable and unique genes were identified by in-house R scripts. The phylogenetic tree based in gene content and split network for gene content were constructed with SplitsTree4 ^11^ using the GeneContentDistance method ^12^. The SI structure and comparison of seven *V. cholerae* and, their sister species, *V. mimicus* were performed using genoPlotR ^13^.

**Table 1.** Superintegron regions extracted from *V. cholerae* and *V. mimicus* genomes.

Organism	Serogroup/ Biotype	Geographical origin	Source of isolation	Year of isolation	Start	End*	Size (bp)	G+C (%)	ORFs	Locus *IntI4*	Accession in NCBI
*V. cholerae* N16961	O1 El Tor	Bangladesh	Clinical	1975	309750	435418	125669	42.20	166	VCA0291	NC_002506
*V. cholerae* 2010EL1786	O1 El Tor	Haiti	Clinical	2010	36195	135658	99464	42.08	138	Vch1786_II0037	NC_016446
*V. cholerae* MJ-1236	O1 El Tor	Matlab, Bangladesh	Clinical	1994	931735	1050596	118862	41.46	135	VCD_000984	NC_012667
*V. cholerae* O395	O1 Classical	India	Clinical	1965	799827	916350	116524	41.35	175	VCO395_0938	NC_009456
*V. cholerae* LMA3984	O1	Para, Brazil	Environmental	2007	294428	332847	38420	42.70	47	VCLMA_B0259	NC_017269
*V. cholerae* M66-2	O1	Indonesia	Clinical	1937	310949	409433	98485	42.15	133	VCM66_A0290	NC_012580
*V. cholerae* IEC224	O1	Para, Brazil	Clinical	1990s	309717	435237	125521	42.21	167	O3Y_14823	NC_016945
*V. mimicus* MB-451	ND	Matlab, Bangladesh	Clinical	ND	744870	872905	128036	41.39	115	VII_000636	NZ_ADAF01000002

*Nucleotide position on the chromosome. ND, not determined.

## Results and discussion

**Figure 2.**
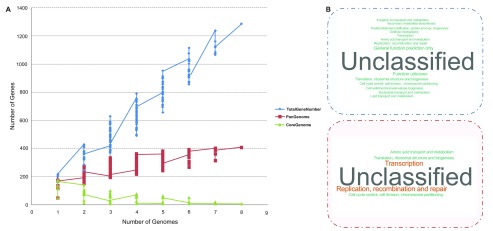
( **A**) Pangenome plot of the SI region considering seven *V. cholerae* and one *V. mimicus* genomes. 1285 total genes, 408 pangenome clusters and eight core clusters were identified. ( **B**) Word clouds of cluster function enrichment comparison according to clusters of orthologous groups (COG) for whole and core clusters identified are shown at the top and bottom, respectively. Clusters that are not assigned in the COG classification were excluded from the figure.

SI regions were extracted from the seven *V. cholerae* and one *V. mimicus* genomes ( Table 1). The 1285 genes recovered were clustered and a total of 408 clusters were detected ( Figure 2A; Table S1). The pangenome of the SI of *Vibrio* strains evaluated was 408 genes, of which eight correspond to core genes, 196 are distributed or dispensable genes and 204 are unique genes. Six of the eight core genes are present in many copies ( Table 2). The pangenome profile analysis shows that the cluster numbers of core genome are almost the same, when the SI considered reaches nine, while the pangenome is still increasing ( Figure 2A). We infer that the *V. cholerae* SI has an open pangenome, which means that *V. cholerae* may have the ability to import new SI gene cassettes, which affect its plasticity and diversity. On the other hand, the set of SIs, from clinical and environmental lineages, used in this study are apparently representative of this species because allowed to establish that the core genome is close to being completed.

**Table 2.** Core genes of the *V. cholerae* SI. The table shows the clusters, conservation level between genomes, the functional categories, gene description and the corresponding locus tag in the reference N16961 genome.

ClusterID	Conservation level	COG*	Description	Locus_tag in N16961
1	8	-	hypothetical protein	VCA0407,VCA0353,VCA0336, VCA0297,VCA0302
2	8	COG0456R	acetyltransferase	VCA0470
3	8	-	lipoprotein	VCA0425,VCA0414
4	8	-	hypothetical protein	VCA0381,VCA0435,VCA0357, VCA0306
5	8	-	hypothetical protein	VCA0434,VCA0411
7	8	COG4974L	site-specific recombinase IntI4	VCA0291
8	8	-	relB protein	VCA0349,VCA0504
9	8	COG1670J	acetyltransferase	VCA0505,VCA0436,VCA0417, VCA0316
24	7	COG0110R	acetyltransferase	VCA0473
25	7	COG3668R	plasmid stabilization element ParE	VCA0359
27	7	COG2944K	virulence gene repressor RsaL	VCA0469
31	7	-	hypothetical protein	VCA0497
32	7	COG1694R	mazG-related protein	VCA0485
33	7	-	cytotoxic translational repressor of toxin- antitoxin stability system	VCA0468
34	7	COG0346E	glyoxalase/bleomycin resistance protein	VCA0506,VCA0347
35	7	-	hypothetical protein	VCA0486
37	7	COG2161D	antitoxin of toxin- antitoxin stability system	VCA0477
40	7	COG0456R	GCN5-related N-acetyltransferase	VCA0382
41	7	COG1943L	IS1004 transposase	VCA0493
43	7	COG3668R	plasmid stabilization system protein	VCA0489
44	7	COG3636K	hypothetical protein	VCA0498

*COG: Cluster of Orthologous Groups; "-" depicts no COG assignation.

Function enrichment analysis of gene clusters were performed according to description of gene annotation ( File S1) supplied to the pangenome analysis pipeline ^10^. From the 408 clusters, 329 were unclassified by the function enrichment analysis. Following the categorization of Cluster of Orthologous Groups (COG), the characterized clusters were rich in the following categories: translation, ribosomal structure and biogenesis, transcription, replication, recombination and repair, cell cycle control, cell division, chromosome partitioning, defense mechanisms, cell wall/membrane/envelope biogenesis and posttranslational modification, protein turnover, chaperones, amino acid transport and metabolism, nucleotide transport and metabolism, lipid transport and metabolism, secondary metabolites biosynthesis, transport and catabolism ( Figure 2B).

**Figure 3.**
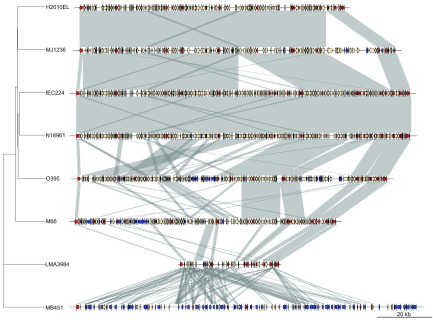
Superintegron (SI) structure and comparison of seven strains of *V. cholerae* and a strain of *V. mimicus*. The core, dispensable and unique genes are indicated by red, cream and blue arrows, respectively. Vertical blocks between sequences indicate regions with more than 1 kb of shared similarity shaded according to BLASTn. A phylogenetic tree based on gene content of the SI is shown on the left.

In the SI, random excisions occur throughout the cassette array to form nonreplicative circular intermediates containing one or several cassettes; integration events preferentially occur at the *attI* site ^5^ and are subjected to selection. It is expected that SI core genes would be arranged and stay together; however, we found the core genes are distributed along the SI ( Figure 3), apparently without any position effect.

We identified 204 unique genes, 94 belonging to *V. mimicus* MB451, nine to LMA3984, 45 to O395, nine to 2010EL1786, 14 to MJ1236, seven to IEC224, 20 to M66, and six to N16961 ( Figure 3; Table S1). Considering only the *V. cholerae* SI, there are 21 core genes, most of them present in many copies and rich in the transcription, replication, recombination and repair, translation, ribosomal structure and biogenesis categories.

Pandey and Gerdes ^14^ identified 13 TA loci within the SI of the N16961 strain. Here we identified six TA genes as part of core SI genes ( Table 2), of which the *relB* genes (VCA0349 and VCA0504) were present in all *V. cholerae* strains (including *V. mimicus*) SIs. The *parE* (VCA0359), *relB* (VCA0477) and *relE* (VCA0489) genes were present in all *V. cholerae* SIs. Moreover, we also identified two *higBA* loci (VCA0469 and VCA468), which encode mRNA cleaving enzymes and can stabilize plasmids ^6^, as well as SI genes. The previous authors ^14^ also identified *higBA-1* TA loci (VCA0392 and VCA0391); in our results, these two TA loci are present in all clinical *V. cholerae* strains ( Table S1). These results suggest that *V. cholerae* TA loci function as essential stress response elements that help cells survive ^6^, as well as act to stabilize the massive arrays of SI cassettes, as reported previously ^15^.

A previous study suggested that SI structure is influenced by geographic boundaries in response to environmental conditions ^8^. Here, we found that the clinical nature of the *V. cholerae* and *V. mimicus* strains evaluated were not grouped together by the analyses performed. Therefore, the ability of *V. cholerae* to cause disease must be explained by other virulence factors found outside the SI region.

**Figure 4.**
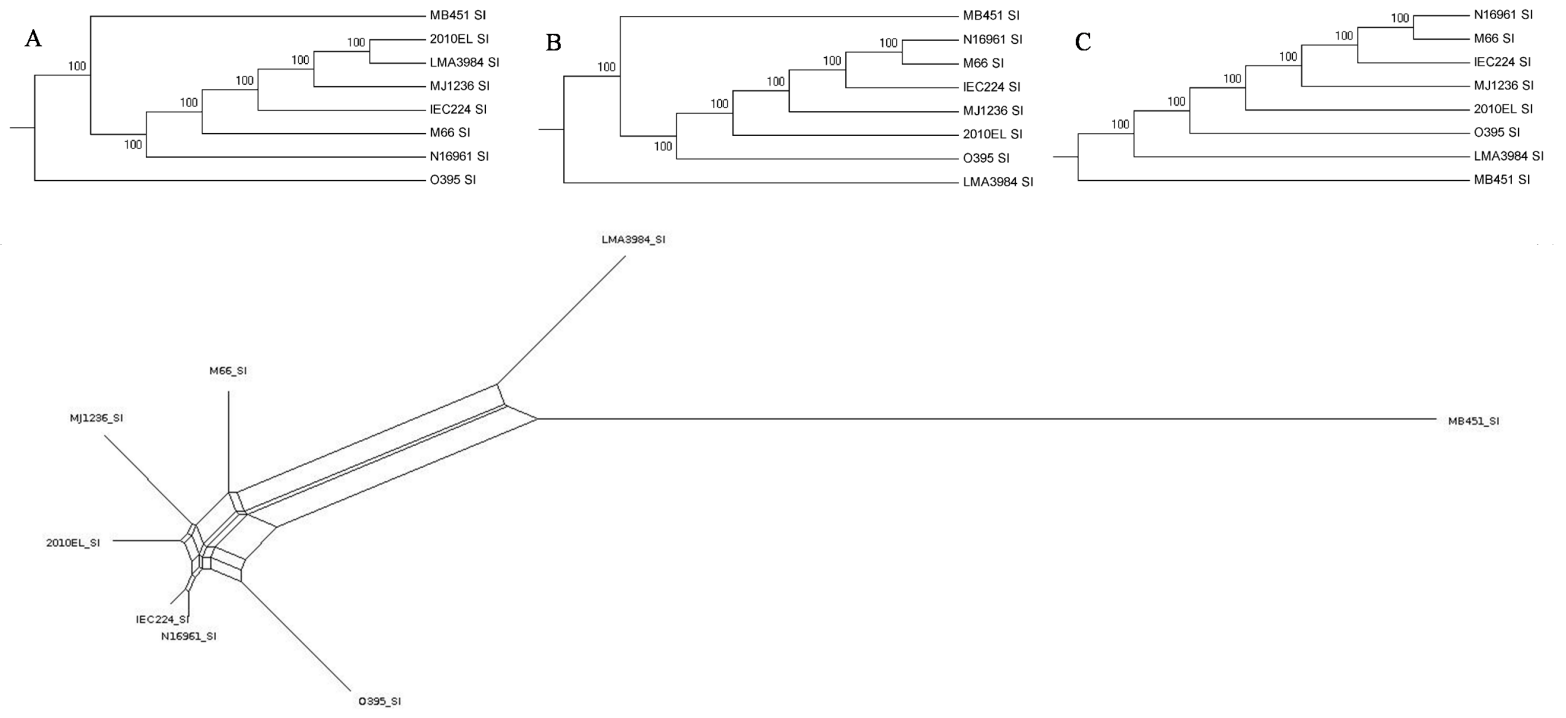
Top: Phylogenetic trees for the *V. cholerae* SI based on SNPs constructed by the Maximum Likelihood ( **A**), Neighbor-Joining ( **B**) and UPGMA ( **C**) methods. The numbers indicate the bootstrap values. Bottom: Split network for gene content based on the 408 genes in seven *V. cholerae* and one *V. mimicus* genomes. The network was constructed with SplitsTree4 using the GeneContentDistance method ^12^.

There are 199 clusters involved with indel or mutation events ( Table S2). As for the non-synonymous/synonymous substitution (dN/dS) ratio, we found that 30 clusters were suffering positive selection pressure (dN/dS > 1). At the same time, we could also select those variable clusters as the markers for different strains. Based on pangenome profiles and single nucleotide polymorphism (SNP) information, gene content and phylogenetic trees were constructed ( Figure 4). The SNP information from SI was useful for separating *V. cholerae* from *V. mimicus*, but nevertheless lacked the resolution to distinguish between the different lineages of *V. cholerae*. However, using gene content information ( Figure 4), a good resolution was reached that was coherent with the evolution of the species and the environmental or clinical nature of the strains. These results indicate that the evolution of *V. cholerae* into different lineages is reflected in the diversity of the SI, which would be also influenced by horizontal gene transfer in these region, as proposed elsewhere ^8, 16, 17^.

File S1. Gene data from V. cholera and V. mimicus. Gene data of SI region from seven V. cholerae and one V. mimicus genomes used in this study.

File S2. Identifying core genome, dispensable and unique genes of superintegron (SI) with R. R scripts used in this study.

Table S1. Orthologs clusters. Orthologs clusters identification among SIs from V. cholerae and V. mimicus genomes. These clusters were identified using the pangenome analysis pipeline (10), strains without genes in the cluster are marked with "-".

Table S2. Clusters involved with indel or mutation events. Clusters involved with indel or mutation events. The 1st column is the Cluster ID, which is consistent with the ID in Table S1. The 2nd column is the cluster conservation of current cluster. The 3rd column is the variation position, which counts according to the alignment result of protein sequences in this cluster. For indel events, the position is an integer. For synonymous mutation and non synonymous mutation, the position is a floating number, in which the integer part marks the position of the amino acid in the alignment result of protein sequences, while the decimal part mark the position of codon. The 4th column shows the amino acid types on current position. The 5th column shows the nucleotide types on current position, indel is marked with "-". The 6th column shows all gene nucleotide profile in current position (for indel, amino acid will be listed). The 7th column shows the variation type (indel, synonymous and non synonymous). The CDS.variation.analysis spreadsheet shows the summary result for CDS.variation.

## Conclusions

In this study, we have revealed the genetic architecture of the *V. cholerae* SI, which contains eight core genes, many of them present in many copies. The *V. cholerae* SI has an open pangenome, which means that *V. cholerae* may have the ability to import new gene cassettes into the SI. The set of the dispensable SI gene cassettes is influenced by the niche and type species. The core genes are distributed along the SI, apparently without a position effect.

## Supplementary tables

Table S1. Orthologs clusters identification among SIs from *V. cholerae* and *V. mimicus* genomes. These clusters were identified using the pangenome analysis pipeline ^10^, strains without genes in the cluster are marked with "-".

Table S2. Clusters involved with indel or mutation events. The 1 ^st^ column is the Cluster ID, which is consistent with the ID in Table S1. The 2 ^nd^ column is the cluster conservation of current cluster. The 3 ^rd^ column is the variation position, which counts according to the alignment result of protein sequences in this cluster. For indel events, the position is an integer. For synonymous mutation and non synonymous mutation, the position is a floating number, in which the integer part marks the position of the amino acid in the alignment result of protein sequences, while the decimal part mark the position of codon. The 4 ^th^ column shows the amino acid types on current position. The 5 ^th^ column shows the nucleotide types on current position, indel is marked with "-". The 6 ^th^ column shows all gene nucleotide profile in current position (for indel, amino acid will be listed). The 7 ^th^ column shows the variation type (indel, synonymous and non synonymous). The CDS.variation.analysis spreadsheet shows the summary result for CDS.variation.

## Supplementary files

File S1. Gene data of SI region from seven *V. cholerae* and one *V. mimicus* genomes used in this study.

File S2. R scripts used in this study.
