# Supplementary material for: Architecture of the superintegron in Vibrio cholerae: identification of core and unique genes
Source: F1000Res. 2013 Feb 27;2:63. [Version 1] doi: 10.12688/f1000research.2-63.v1 (PMC4032106; doi:10.12688/f1000research.2-63.v1)
Supplement: Peer review history

# Peer review: Referee response for version 1

**Published:** 2014-04-14

**Authors:** Nur Hasan

**Affiliations:** 1 Maryland Pathogen Research Institute, University of Maryland, College Park, MD, USA

In this manuscript Marin and Vicente investigated the genomic diversity of *V. cholerae* Super Integron (SI) with the aim to identify a set of orthologous genes that are conserved and unique among and in between *V. cholerae* and *V. mimicus *SI’s. While one must appreciate the efforts that have gone into the analyses, unfortunately, given the known diversity of *V. cholerae* SI, the number of genomes analyzed was very limited, and was not a good representative of all major phyletic lineages of *V. cholerae *either. Yet, the manuscript provides some valuable information about the diversity and repository of SI genes and their biological functions.

The core genes estimates for SI (21 and 8, among *V. cholerae* and *V. cholerae-V. mimicus* respectively) reported in this study may not be very meaningful as the core genome might diminish or at least reduced further if additional genomes from distinct lineages are included. I strongly recommend inclusion of additional genomes at least from the major phylogenetic lineages of *V. cholerae* O1.

The function of TA loci as an essential stress response element needs to be supported by some experimental data.

I have read this submission. I believe that I have an appropriate level of expertise to confirm that it is of an acceptable scientific standard.

# Peer review: Referee response for version 1

**Published:** 2014-03-14

**Authors:** Yan Boucher

**Affiliations:** 1 Department of Biological Sciences, University of Alberta, Edmonton, AB, Canada

The article, although it has a very narrow focus, investigates an interesting question about integron regions in *V. cholerae* that scientists have so far mostly applied to whole genome sequences: what is their pan-genome and core genome? The scope of the question is quite narrow, as it focuses on a single species ( *V. cholerae*), but is certainly novel.

The methods and approach used are sound and the results generally well presented. I have two major issues with the analysis:

1. Why use only seven genomes when >200 are available? This is quite puzzling to me, as no data should be excluded if it is available. Some of the >200 *V. cholerae* genomes available are relatively redundant (they all belong to the 7 ^th^ pandemic group) and present little gene content/SNP diversity, but the variations they present are crucial in understanding small scale variability. Many available environmental *V. cholerae *genomes were also not included and should have been.
2. The article is mostly descriptive and needs to make more informative statements about the results. What is the significance of such a small core genome? How does the pan-genome of the integron overlap with the rest of the genome? What is the meaning of the specific subset of functions found in the integron? How are sites under positive selection distributed in the integron? What genes are under positive/ negative selection? Much more can be said about integrons from the analyses performed.

Basically, the manuscript is interesting but could really benefit from a broader (more genomes) analysis and a more in-depth look at the results to infer hypotheses about integrons in *V. cholerae*.

I have read this submission. I believe that I have an appropriate level of expertise to confirm that it is of an acceptable scientific standard, however I have significant reservations, as outlined above.

# Peer review: Referee response for version 1

**Published:** 2013-05-09

**Authors:** Thandavarayan Ramamurthy

**Affiliations:** 1 National Institute of Cholera and Enteric Diseases, Kolkata, India

In this manuscript the authors have explored the pattern of the superintegrons (SIs) in *V. cholerae* using the published DNA sequences of relevant strains. This study has shown the dynamic nature of *V. cholerae* O1 and genetic relatedness of SIs at the biotype level.

Comments:

- Abstract: Results and Conclusions: replace the word ‘reveal’ with “describe”
- Introduction: first paragraph: replace the word ‘standing’ with “standby”
- Page 6. The role of TA gene should be validated with non-toxigenic strains of *V. cholerae *(e.g., sequence comparison with non-O1, non-O139 strains of *V. cholerae*

I have read this submission. I believe that I have an appropriate level of expertise to confirm that it is of an acceptable scientific standard.
